# Associated deaths and disability-adjusted life-years caused by infections with antibiotic-resistant bacteria in Switzerland, 2010 to 2019

**DOI:** 10.2807/1560-7917.ES.2023.28.20.2200532

**Published:** 2023-05-18

**Authors:** Michael Gasser, Alessandro Cassini, Danilo Lo Fo Wong, Marcello Gelormini, Saskia Andrea Nahrgang, Walter Zingg, Andreas Oskar Kronenberg

**Affiliations:** 1 Swiss Centre for Antibiotic Resistance (ANRESIS), Institute for Infectious Diseases, University of Bern, Bern, Switzerland; 2 Deputy Cantonal Doctor, Public Health Department, Canton of Vaud, Lausanne, Switzerland; 3 Infection Prevention and Control Unit, Infectious Diseases Service, Lausanne University Hospital, Lausanne, Switzerland; 4 Control of Antimicrobial Resistance Programme, World Health Organization Regional Office for Europe, Copenhagen, Denmark; 5 Division of Infectious Diseases and Hospital Epidemiology, University Hospital Zurich, Zurich, Switzerland

**Keywords:** Antimicrobial resistance (AMR), burden of disease, disability-adjusted life-years (DALYs), regional stratification

## Abstract

**Background:**

Cassini et al. (2019) estimated that, in 2015, infections with 16 different antibiotic-resistant bacteria resulted in ca 170 disability-adjusted life-years (DALYs) per 100,000 population in the European Union and European Economic area (EU/EEA). The corresponding estimate for Switzerland was about half of this (87.8 DALYs per 100,000 population) but still higher than that of several EU/EEA countries (e.g. neighbouring Austria (77.2)).

**Aim:**

In this study, the burden caused by the same infections due to antibiotic-resistant bacteria (‘AMR burden’) in Switzerland from 2010 to 2019 was estimated and the effect of the factors ‘linguistic region’ and ‘hospital type’ on this estimate was examined.

**Methods:**

Number of infections, DALYs and deaths were estimated according to Cassini et al. (2019) whereas separate models were built for each linguistic region/hospital type combination.

**Results:**

DALYs increased significantly from 3,995 (95% uncertainty interval (UI): 3;327–4,805) in 2010 to 6,805 (95% UI: 5,820–7,949) in 2019. Linguistic region and hospital type stratifications significantly affected the absolute values and the slope of the total AMR burden estimates. DALYs per population were higher in the Latin part of Switzerland (98 DALYs per 100,000 population; 95% UI: 83–115) compared with the German part (57 DALYs per 100,000 population; 95% UI: 49–66) and in university hospitals (165 DALYs per 100,000 hospitalisation days; 95% UI: 140–194) compared with non-university hospitals (62 DALYs per 100,000 hospitalisation days; 95% UI: 53–72).

**Conclusions:**

The AMR burden estimate in Switzerland has increased significantly between 2010 and 2019. Considerable differences depending on the linguistic region and the hospital type were identified – a finding which affects the nationwide burden estimation.

**Key public health message**

**What did you want to address in this study?**

We wanted to assess the burden (i.e. deaths/other parameters) of infections with antibiotic-resistant bacteria in Switzerland in 2010–2019. In this period, the burden overall, and the burden each year, were estimated for the country. We also explored how hospital types and linguistic regions might differently contribute to the national estimate. Last, we looked if measures taken to control resistant pathogens might have alleviated the problem.

**What have we learnt from this study?**

The burden of infections by antibiotic-resistant bacteria in Switzerland increased significantly between 2010 and 2019, but to a moderate level compared to the European average. Within the country, burden estimations differed by linguistic region and hospital types. These factors considerably impacted the overall burden estimation at national level. Amounts of highly resistant pathogens stayed relatively low while measures were implemented.

**What are the implications of your findings for public health?**

We found that considering linguistic regions and hospital types in the burden calculations makes it possible to identify regional deficits that need to be addressed. Furthermore, taking these factors into account improved the accuracy of burden estimates at national level. This finding may also encourage other countries to adopt such an approach, allowing for more accurate comparisons between countries.

## Introduction

Estimates of the impact of infectious diseases are needed for an accurate risk assessment, as well as for planning and prioritising public health resources. Disability-adjusted life-years (DALYs) [1] are a widely used measure of the overall disease burden accounting for healthy life years lost because of premature mortality and years lost living with disabilities for each condition or disease. Cassini et al. estimated that ca 672,000 infections, 33,100 deaths and 875,000 DALYs resulting from infections with 16 antibiotic resistance (AMR)–bacterium combinations occurred in the European Union (EU) and European Economic Area (EEA) in 2015 [2]. By applying the same approach, ca 7,160 infections, 276 associated deaths and 7,400 DALYs were estimated for Switzerland in 2015 [3]. A comparison with individual EU and EEA countries revealed that the 2015 estimate for Switzerland (87.8 DALYs per 100,000 population) was higher than those in neighbouring Austria (77.2) or Germany (64.3) but considerably lower than adjacent Italy (448.4) or France (220.7). Compared with other countries with similar economic performance indicators such as Luxembourg (70.9 DALYs per 100,000 population), Denmark (52.3) or Norway (33.1), the estimate for Switzerland was also rather high, whereas in a Europe-wide comparison (EU/EAA median 170 DALYs per 100,000 population) it was rather low [2,3].

Published studies [2,3] have focused on (supra)national estimates in one or two time points. The main aim of the current investigation was to update the estimate of the burden of disease due to infections with antibiotic-resistant bacteria in 2019 and analyse the epidemiological trend since 2010. Due to well-documented differences in resistance patterns depending on the hospital types (i.e. university vs non-university hospitals) and linguistic regions in Switzerland [4-6] analyses were stratified by these factors. An additional objective was to qualitatively explore whether measures to curb AMR in Switzerland [7] might have somewhat affected DALYs.

## Methods

### Data retrieval and estimation of infections

The COVID-19 pandemic may have increased or decreased the burden of antimicrobial resistance [8]. To avoid interference, this study was restricted to the years before the pandemic (2010–2019).

The methodology from Cassini et al. [2] was adopted and the same 16 AMR–bacterium combinations were included (Table). Data from blood and cerebrospinal fluid (invasive isolates, hereinafter referred to as BSIs) were obtained from the Swiss Centre for Antibiotic Resistance (ANRESIS) national database. Data were deduplicated by keeping only the first isolate of a given microorganism per patient per year. Aggregations were performed by the resistance–bacterium combination, age group (categorical variable, i.e. 0–1, 2–4, 5–9, 10–14, …, 80–84, ≥ 85 years) and sex (binary variable, i.e. male/female). Unknown age and sex data were redistributed by imputation. As the hospitalisation date was not available for most infections, no distinction was made between community and hospital-acquired infections.

**Table:** Bacteria and antibiotic resistance categories included in the study^a^, Switzerland, 2010–2019

Bacteria	Antibiotic resistance^b^	Acronym
***Acinetobacter* spp.**	Colistin-resistant	ColRACI
	Carbapenem-resistant (excluding isolates also resistant to colistin)	CRACI
	Aminoglycoside- and fluoroquinolone-resistant^c^ (excluding isolates also resistant to colistin and/or carbapenem)	MDRACI
***Enterococcus faecalis* and *E. faecium***	Vancomycin-resistant	VRE
***Escherichia coli***	Colistin-resistant	ColREC
	Carbapenem-resistant (excluding isolates also resistant to colistin)	CREC
	Third-generation cephalosporin-resistant (excluding isolates also resistant to colistin and/or carbapenem)	3GCREC
***Klebsiella pneumoniae***	Colistin-resistant	ColRKP
	Carbapenem-resistant (excluding isolates also resistant to colistin)	CRKP
	Third-generation cephalosporin-resistant (excluding isolates also resistant to colistin and/or carbapenem)	3GCRKP
***Pseudomonas aeruginosa***	Colistin-resistant	ColRPA
	Carbapenem-resistant (excluding isolates also resistant to colistin)	CRPA
	Resistance to three or more antibiotic groups^c^ (excluding isolates also resistant to colistin and/or carbapenem)	MDRPA
***Staphylococcus aureus***	Meticillin-resistant	MRSA
***Streptococcus pneumoniae***	Penicillin-resistant (excluding isolates also resistant to macrolides)	PRSP
	Penicillin- and macrolide-resistant (excluding isolates only resistant to penicillin)	PMRSP

EARS-Net: European Antimicrobial Resistance Surveillance Network.

^a^ Adapted from Cassini et al. [2].

^b^ An isolate was considered resistant to an antimicrobial group when tested and interpreted as resistant (R) in accordance with the clinical breakpoint criteria used by the local laboratory.

^c^ Resistances used as a marker of multidrug resistance.

For more information on the antibiotics included in each antibiotic group, please refer to the EARS-Net protocol [37].

BSIs are the most completely reported infections in Switzerland, while other infections are less fully reported. Ratios of BSIs to non-BSIs (conversion factors) for each AMR–bacterium combination derived from the European Centre for Disease Prevention and Control (ECDC) point prevalence survey (PPS) 2016–2017 [9] were therefore used to estimate the number of urinary tract infections, respiratory tract infections, surgical site infections, and other infections. In the PPS 2016–2017, infection site incidence for each resistance–bacterium combination was calculated by applying the Rhame and Sudderth formula to the prevalence data [10]. Uncertainty around the conversion factors was assessed using bootstrap resampling. The number of estimated non-BSIs (urinary tract infections, etc.) was then added to the number of BSIs to obtain the total number of infections.

The percentage of secondary BSIs from the PPS 2016–2017 was deducted from each of the non-BSIs in the Swiss dataset as described in [2]. To automatise different data processing steps, parametrised workflows were built using the KNIME Analytics Platform version 4.3.1.

### Data analysis

For the classification of data according to the linguistic region, the French and the Italian speaking parts are grouped as ‘Latin part’. The German-speaking part is referred to as ‘German part’ for simplicity. Demographic data were obtained from the Swiss Federal Statistical Office [11].

For the main analysis (hereinafter referred to as ‘first analysis’) coverage correction factors for different hospital types (university vs non-university) and for different linguistic regions were calculated using the number of hospitalisation days [12] of the comprised hospitals (see Supplementary Table S1 for different coverage rates). Numbers of infections found at each stratification level were added after applying the coverage correction factors to get the total number of infections for the whole country. These findings were then compared with two additional models (‘second’ and ‘third analysis’). In both models, coverage correction factors were calculated yearly for the whole country without any further stratification. In the second analysis data from all hospitals included in ANRESIS were used, while in the third analysis the dataset was restricted to hospitals, that have been reporting to ANRESIS since 2010.

In contrast to Cassini et al. [2], the term ‘associated infections’ is used in this study instead of ‘attributable infections’ to express that estimations are based on a scenario in which all drug-resistant infections were replaced by no infection, rather than on a scenario in which all drug-resistant infections were replaced by susceptible ones. This terminology is in accordance with a recent study from Murray et al. [13].

DALYs and associated deaths were estimated according to Cassini et al. [2] using the ECDC BCoDE toolkit Version 2.0.0 [14] based on 10,000 Monte Carlo simulations, without time discounting. Uncertainties of conversion factors were included in the models as PERT distributions and disease model parameters were given with 95% uncertainty intervals (UIs). Medians of the distributions were used as point estimates. When aggregating output values, two different proceedings were applied to calculate the corresponding UIs. In one approach a 100% positive correlation between sampled values was assumed and UIs were summed; in another approach total independence was assumed and UIs were approximated by the square root of the sum of squares. Hereinafter only results from the first more conservative proceeding are presented (see Supplementary Figure S7 for two examples using the second approach).

The R software environment (version 4.0.4) was used to execute the BCoDE toolkit and for analyses and visualisations, which were performed with the BCoDE output parameters [15].

### Measures to curb antibacterial resistance

**Figure 1:**
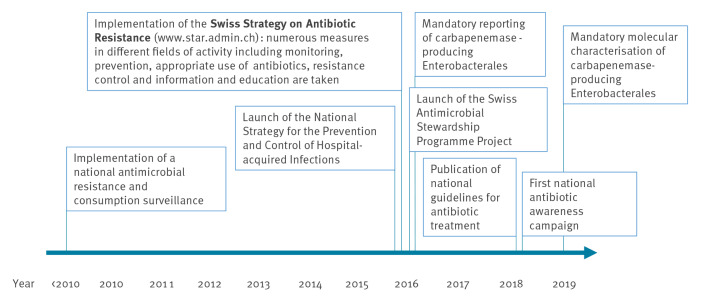
Some measures to prevent the development of resistant bacteria and limit their transmission and spread in Switzerland, prior and during the 2010–2019 study period

During the study period, several measures were implemented in Switzerland to curb AMR. Most of them were bundled in the Swiss Strategy on Antibiotic Resistance (StAR), which was implemented as of 2016 [7]. A qualitative comparison of these measures with annual AMR burden estimates was discussed in terms of the effectiveness of such interventions. A selection of important measures is schematically represented in Figure 1.

## Results

### First analysis – estimation of the burden of infections with antibiotic-resistant bacteria stratified by linguistic region and hospital type

A total of 5,610 BSIs were counted for the study period (before applying country coverage factors). The patient’s age was available for all isolates, and information about the sex for 5,608 (99.96%).

#### Distribution of the burden by year across the study period

**Figure 2:**
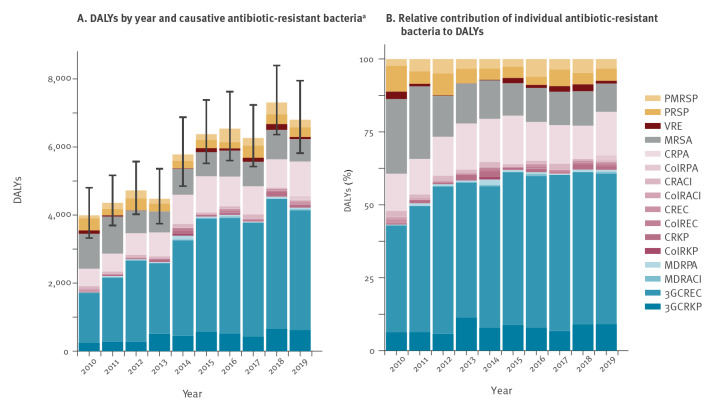
(A) Disability-adjusted life-years (DALYs) caused by infections with antibiotic-resistant bacteria^a^ and (B) relative contributions of individual antibiotic-resistant bacteria to the DALYs, Switzerland, 2010–2019

It was estimated that 3,110 (95% UI: 2,516–3,844) infections due to antibiotic-resistant bacteria from different locations occurred in 2010 increasing to 6,342 (95% UI: 5,316–7,538) in 2019 (+ 104%; see stacked bar charts of different antibiotic-resistant bacteria in Supplementary Figure S1 and absolute numbers in 2010 and 2019 in Supplementary Table S3). These estimates accounted for 3,995 DALYs (95% UI: 3,327–4,805) in 2010 increasing to 6,805 DALYs (95% UI: 5,820–7,949) in 2019 (+ 70%; see Figure 2A and Supplementary Table S3). Deaths associated with infections due to antibiotic-resistant bacteria increased from 136 (95% UI: 114–161) in 2010 to 286 (95% UI: 243–335) in 2019, corresponding to + 111% (when calculating from the initially non-rounded estimates). This increase is illustrated in Supplementary Figure S2 (see stacked segments in the bar charts corresponding to different antibiotic-resistant bacteria) and absolute values for each year are presented in Supplementary Table S3. These estimates correspond to 40 infections per 100,000 population (95% UI: 32–49) in 2010 and 74 infections per 100,000 population (95% UI: 62–88) in 2019 respectively, 51 DALYs per 100,000 population (95% UI: 42–61) in 2010 and 79 DALYs per 100,000 population (95% UI: 68–92) in 2019 respectively and 1.72 deaths per 100,000 population (95% UI: 1.44–2.05) in 2010 and 3.32 deaths per 100,000 population (95% UI: 2.82–3.89) in 2019 respectively.

ColRACI: colistin-resistant *Acinetobacter* spp.; ColREC: colistin-resistant *Escherichia coli*; ColRKP: colistin-resistant *Klebsiella pneumoniae*; ColRPA: colistin-resistant *Pseudomonas aeruginosa*; CRACI: carbapenem-resistant *Acinetobacter* spp.; CREC: carbapenem-resistant *E. coli*; CRKP: carbapenem-resistant *K pneumoniae*; CRPA: carbapenem-resistant *P. aeruginosa*; MDRACI: multidrug-resistant *Acinetobacter* spp.; MDRPA: multidrug-resistant *P. aeruginosa*; MRSA: meticillin-resistant *Staphylococcus aureus*; PMRSP: penicillin-resistant and macrolide-resistant *S pneumoniae*; PRSP: penicillin-resistant *Streptococcus pneumoniae*; VRE: vancomycin-resistant *Enterococcus faecalis* and *Enterococcus faecium*; 3GCREC: third-generation cephalosporin-resistant *E. coli*; 3GCRKP: third-generation cephalosporin-resistant *K pneumoniae*.

^a^ Absolute values and 95% uncertainty intervals (black bars) of the yearly totals are shown on panel A.

Data which had been stratified according to the linguistic region and the hospital type were aggregated.

Throughout the whole study period, most DALYs were associated with third-generation cephalosporin-resistant *Escherichia coli* (1,461 DALYs (95% UI: 1,294–1,634) in 2010 and 3,511 DALYs (95% UI: 3,187–3,851) in 2019) (Figure 2A and Supplementary Table S3), which contributed to 37% of the total DALYs in 2010 (Figure 2B). After a relatively steep increase until 2013, this value stabilised at around 50% during subsequent years (52% in 2019), resulting in a less pronounced overall increase in DALYs in the later years of the study (Figure 2A). The second biggest contributor to the burden was meticillin-resistant *Staphylococcus aureus* (MRSA) with 1,022 DALYs (95% UI: 898–1,169) in 2010 and 656 DALYs (95% UI: 581–742) in 2019, corresponding to 26% in 2010 and 10% in 2019. As the burden from MRSA continuously decreased during this period, it became surpassed in 2012 by carbapenem-resistant *Pseudomonas aeruginosa* with 510 DALYs (95% UI: 333–739) in 2010 and 1,012 DALYs (95% UI: 735–1,348) in 2019, corresponding to 13% in 2010 and 15% in 2019. Other carbapenem-resistant bacteria remained at a low level throughout the whole study period (e.g. carbapenem-resistant *E. coli* with 53 DALYs (95% UI: 32–76) in 2010 and 51 DALYs (95% UI: 34–69) in 2019, corresponding to 1 % in 2010 and 1 % in 2019). Similarly, all colistin-resistant bacteria remained at a low level or were even absent (see Figure 2A and Figure 2B for their representation in stacked bar charts, Supplementary Table S3 for absolute numbers and https://www.anresis.ch/wp-content/uploads/2022/03/2022_02_bubble_plot_allstrat_agg.html to get a dynamic picture of how numbers of infections, DALYs and deaths from different antibiotic-resistant bacteria evolve over time).

#### Distribution of the burden by age and sex

**Figure 3:**
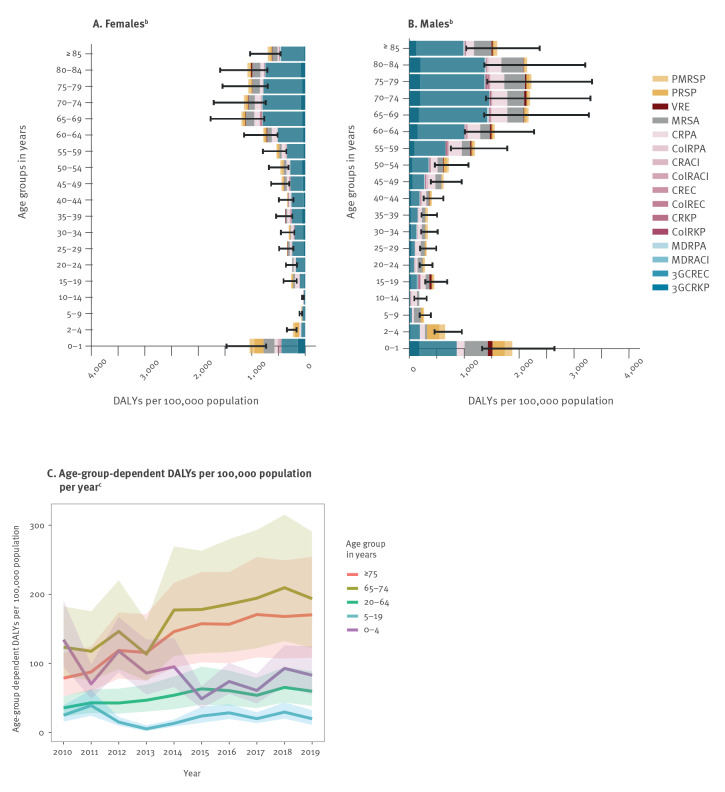
Age-group-dependent model estimates of DALYs^a^ per 100,000 population for the whole study period by (A) female^b^ and (B) male^b^ sex, and (C) for each year of the study^c^, Switzerland, 2010–2019

Males accounted for 62% of the estimated total number of DALYs (2010–2019 pooled data) and the burden in DALYs per 100,000 population was higher compared with females in every age group, with 25–29 and 35–39-year-olds being exceptions (Figure 3A and 3B). In both males and females, the highest values were found in age groups between 65 and 84 years old. In the distribution of the burden by age and sex, a second peak, which was more pronounced in males was observed in neonates and infants up to 1 year old. DALYs per 100,000 population (using age group specific denominators) in up to 4-year-old children were decreasing during the study period from 134 (95% UI: 95–191) in 2010 to 83 (95% UI: 55–125) in 2019 (− 38%, Figure 3C). In contrast, values concerning the elderly population were increasing during that period. For example, for those ≥ 75 years old DALYs per 100,000 population increased from 79 (95% UI: 53–115) to 170 (95% UI: 108–254) (+ 117%; when calculating from the initially non-rounded estimates). Age-specific distributions stratified by linguistic region and hospital type are described in Supplementary Figure S4.

ColRACI: colistin-resistant *Acinetobacter* spp.; ColREC: colistin-resistant *Escherichia coli*; ColRKP: colistin-resistant *Klebsiella pneumoniae*; ColRPA: colistin-resistant *Pseudomonas aeruginosa*; CRACI: carbapenem-resistant *Acinetobacter* spp.; CREC: carbapenem-resistant *E. coli*; CRKP: carbapenem-resistant *K pneumoniae*; CRPA: carbapenem-resistant *P. aeruginosa*; DALYs: disability-adjusted life-years; MDRACI: multidrug-resistant *Acinetobacter* spp.; MDRPA: multidrug-resistant *P. aeruginosa*; MRSA: meticillin-resistant *Staphylococcus aureus*; PMRSP: penicillin-resistant and macrolide-resistant *S pneumoniae*; PRSP: penicillin-resistant *Streptococcus pneumoniae*; VRE: vancomycin-resistant *Enterococcus faecalis* and *Enterococcus faecium*; 3GCREC: third-generation cephalosporin-resistant *E. coli*; 3GCRKP: third-generation cephalosporin-resistant *K pneumoniae*.

^a^ DALYs are caused by infections with antibiotic-resistant bacteria.

^b^ Data from 2010 to 2019 were aggregated for these representations.

^c^ Age-group-dependent DALYs per 100,000 population per year are shown in panel C. Sexes and original age groups were pooled in this representation for an improved visualisation. 95% uncertainty intervals are depicted by semi-transparent ribbons.

#### Distribution of the burden by linguistic regions and hospital type

**Figure 4:**
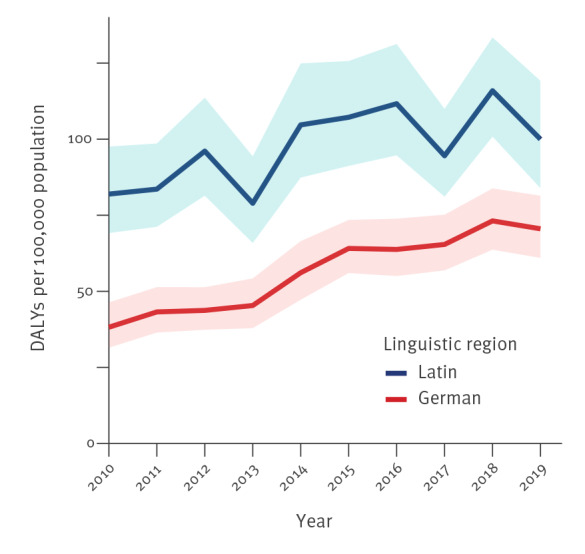
Model estimates of the burden of infections with antibiotic-resistant bacteria of public health importance in DALYs per 100,000 population by linguistic region, Switzerland, 2010–2019

DALYs which were standardised per 100,000 population were higher in the Latin part of Switzerland (98 DALYs per 100,000 population; 95% UI: 83–115) compared with the German part (57 DALYs per 100,000 population; 95% UI: 49–66). Values were increasing in both linguistic regions (Figure 4), however, a higher relative increase (+ 85%) was observed in the German part (Latin part: + 22%). The highest increase was observed in non-university hospitals of the German part (+ 111%) whereas in other settings more moderate increases were observed (university hospitals, German part: + 82%; non-university hospitals, Latin part: + 60%; university hospitals, Latin part: + 15%; Supplementary Figure S5).

DALYs: Disability-adjusted life-years Disability-adjusted life-years.

95% uncertainty intervals are depicted by semi-transparent ribbons.

Infections with antibiotic-resistant bacteria in university hospitals accounted for 41% of DALYs (23,242; 95% UI: 19,673–27,408) over the whole study period, infections in non-university hospitals for 59% of DALYs (33,413; 95% UI: 28,691–38,956). These numbers correspond to 165 DALYs per 100,000 hospitalisation days (95% UI: 140–194) for university hospitals and 62 DALYs per 100,000 hospitalisation days (95% UI: 53–72) for non-university hospitals (denominator data can be found in Supplementary Table S1).

Different distributions of antibiotic-resistant bacteria were found depending on the linguistic region and the hospital type (Supplementary Figure S6). Notably, a higher proportion of DALYs was associated with carbapenem-resistant *P. aeruginosa* in university hospitals (German and Latin parts, both 18%) compared with non-university hospitals (German part 10%, Latin part 12%). In contrast, proportions of DALYs which were associated with third-generation cephalosporin-resistant *E. coli* were lower in university hospitals (German part 42%, Latin part 37%) compared with non-university hospitals (German part 60%, Latin part 50%).

### Comparison of different estimation approaches

**Figure 5:**
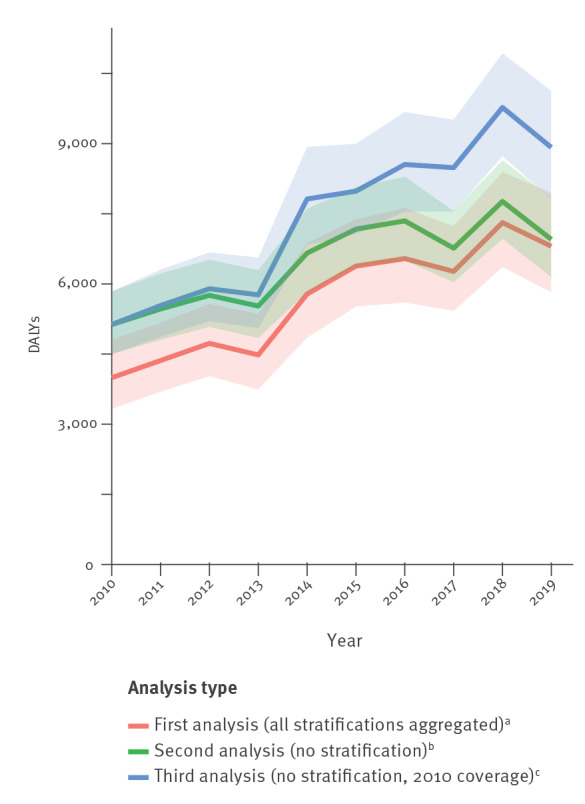
Estimation by three different methodological approaches of DALYs caused by infections with antibiotic-resistant bacteria in Switzerland, 2010–2019

In the second analysis without stratifications by linguistic region and hospital type, it was found that DALYs increased by + 36% (Figure 5). A more pronounced increase (+ 74%) between 2010 and 2019 was observed in the third analysis which was restricted to hospitals, that have been reporting to ANRESIS since 2010.

DALYs: Disability-adjusted life-years Disability-adjusted life-years.

^a^ In the first analysis, the coverages and outputs for each linguistic region and hospital type combination were estimated yearly and all values were then aggregated.

^b^ In the second analysis, the coverage was calculated yearly but no stratification was performed.

^c^ The third analysis included only hospitals which were covered by ANRESIS from the first year of the study (2010) and no stratification was performed.

95% uncertainty intervals are depicted by semi-transparent ribbons.

## Discussion

The availability and quality of BSI data collected since more than a decade by the ANRESIS surveillance system allowed to estimate the burden of infections with 16 AMR–bacterium combinations in Switzerland over an extended period (2010–2019) according to Cassini et al. [2]. Moreover, it was possible to investigate the burden of AMR by hospital type and regional characteristics based on linguistic regions.

The first analysis has shown that the number of infections has more than doubled between 2010 and 2019 leading to a corresponding increase in the number of deaths (+ 111%). The latter observation is comparable with findings from the EU and EEA, where the overall number of deaths increased by a factor of 2.46 however, during a shorter and shifted period (2007–2015) [2]. Interestingly, these high increases in the number of infections and deaths are only partially reflected in the increase of DALYs (+ 70%). This might be due to age group specific long-term trends as shown in Figure 3C. Increases in numbers of infections and deaths were mainly observed in the ≥ 65-year-olds, while decreases were observed in children under 4 years of age during the study period. Thus, given how DALYs are computed (in particular, the years of life lost (YLL) component), this increase in infections and deaths would only marginally increase the number of DALYs.

Several measures (Figure 1), which were implemented by the Swiss authorities, professional societies, healthcare professionals and academia during the last years [7] may have contributed to some stabilisation of the situation after 2015. Besides the implementation of a national surveillance of antimicrobial resistances and consumption as basis for all measures, active intervention strategies were enforced in parallel in different settings. Some interventions which were implemented in the inpatient setting are the introduction of guidelines for the prevention, control and tackling of outbreaks of multidrug-resistant pathogens [16] and for antibiotics use [17], patient screening and isolation of carriers of multidrug-resistant organisms [16,18] as well as the mandatory reporting of carbapenemase-producing Enterobacterales. These measures may have had a strong effect on the number of deaths by keeping infections with high mortality (such as those with microorganisms resistant to carbapenem) at bay, and they may further explain decreasing MRSA rates which have been observed between 2008 and 2021 [19,20] and decreasing DALYs attributed to MRSA infections.

It is important to note that correlating the measures with the available data is difficult, as several measures were bundled and implemented differently in individual cantons due to Switzerland's federal system.

Unfortunately, so far, interventions in the outpatient sector like the implementation of national guidelines for the appropriate use of antibiotics or public campaigns [7] were only partially able to prevent the increase in DALYs from third-generation cephalosporin-resistant *E. coli*, an important pathogen in the outpatient sector [21]. A similar situation was described by Cassini et al. for the EU and EEA, where a strong increase in DALYs associated with these pathogens was observed between 2007 and 2015, contributing the highest proportion of DALYs [2]. Interestingly, the increase in Switzerland was most prominent in people ≥ 65 years old, a finding which merits closer analysis.

In line with previous studies [22,23], it is not surprising that the burden was estimated to be higher in males compared with females. Explanations for sex-dependent resistance patterns are diverse and complex and include both biological and socio-cultural aspects [23,24]. Remarkably, the burden was higher in females than in males in age classes of 25–29 and 35–39 years old, mainly due to infections with third-generation cephalosporin-resistant Enterobacterales. This observation may be explained by increased incidences of urinary tract infections in younger women or infections during pregnancies and childbirths in these cohorts [25].

DALYs per 100,000 population were higher in the Latin part of Switzerland, which borders France and Italy compared with the German part bordering Germany. As burden estimates for Italy and France are higher than those for Germany [2] this finding may be explained by the cross-border movement of individuals, including patients and medical staff between neighbouring countries. This effect may be particularly relevant in the Latin part as numbers of cross-border commuters are considerably higher in this area than in the larger German part [26]. Centrally located in continental Europe, Switzerland is a highly connected country and similar incidence rates and even genetic characteristics of antibiotic-resistant bacteria in neighbouring countries and the bordering regions of Switzerland have been reported previously [5,6]. Another relevant factor may be the region-specific levels of antibiotic consumption. While in 2019 total antibiotic consumption in hospital care was relatively similar in different linguistic regions, considerably higher numbers were observed in the Latin part compared with the German part in the outpatient setting [19]. Filippini et al. hypothesised that these differences may also be explained by cultural variations [27], i.e. that public perception and ‘medical attitudes’ may be influenced by those in neighbouring countries.

It was not unexpected that numbers of DALYs which were standardised by the hospitalisation days were significantly higher for university-hospitals as these hospitals traditionally accommodate more complex cases. Remarkably, the increase in estimated DALYs was more pronounced in non-university hospitals of the German part than in any other setting. This observation may be driven by large high-end cantonal hospitals, mainly located in the German part, which are increasingly treating complex cases, as suggested by an increasing number of intensive care unit (ICU) patients in non-university hospitals (ANRESIS internal data).

Comparisons of different analysis types (Figure 5) show an increase in the number of DALYs for all three approaches. However, the model configuration (i.e. the selection of hospitals and the stratification) has a remarkable effect on both the slope and the level of DALYs time series. The approach using yearly calculated hospital coverage correction factors but without stratifications according to the hospital type and the linguistic regions probably leads to an overestimation of the burden in the early years of the study as proportionally more data from larger university hospitals were available at this time (see Supplementary Table S1 for coverage rates and Supplementary Table S2 for medians and means of hospitalisation days per hospital type). Similarly, de Kraker et al. reported that tertiary care hospitals which may harbour more resistant strains more likely participate in surveillance programmes than smaller hospitals [28] – a statement, which may be particularly relevant at an early stage of the implementation of a surveillance programme. Such a bias may be levelled out in this study in the later years when the surveillance coverage improved and increasingly more non-university hospitals were providing data. Thus, the less pronounced increase, which arises from the unstratified approach has probably no epidemiological causation and may lead to an over-optimistic prognosis for the years to come. In the third analysis, where the extrapolation was restricted to hospitals, that have been reporting to ANRESIS since 2010 the aforementioned effect may be observed throughout the whole study period i.e. the inclusion of larger hospitals with more severe cases may have led to a constant overestimation of the burden. Thus, by stratifying by the hospital type and the linguistic region an overestimation bias may be reduced throughout the whole study period. This bias may become particularly relevant when the burdens of different countries differing in coverage rates are compared – especially countries with national surveillance systems at an early implementation stage, which mainly include higher level-of-care hospitals.

Of note, in the preceding study [3] estimating the 2015 AMR burden for Switzerland no stratification was used. If these estimates are compared with the equivalent estimates (i.e. the ‘no stratification’ approach) of the current study, values are relatively close to each other (7,400 DALYs in the preceding study vs ca 7,200 DALYs in the current study). A small residual difference remains, due to the different point prevalence studies which were used [9,29] and due to the variation from the Monte Carlo simulations.

To our knowledge only one similar study exists estimating the AMR burden for Switzerland. Mestrovic et al. [30] used the approach of Murray et al. [13] to estimate the associated and the attributable deaths for the whole World Health Organization (WHO) European region and for each individual country. For Switzerland they estimated 149 attributable (i.e. comparing with a scenario where infections with resistant pathogens are replaced with susceptible ones) and 738 associated (i.e. comparing with a scenario where drug-resistant infections would not occur at all) deaths for 2019 using the 11 antibiotic-resistant bacteria which were considered in both studies. The latter value is higher compared with the estimate of 278 associated deaths for the same 11 antibiotic-resistant bacteria obtained in this study in 2019. However, a direct comparison is difficult because the counterfactual scenarios are not exactly the same and the model architectures of the two approaches are completely different.

As the method of Cassini et al. [2] was applied, most of the limitations which are described in detail in their study (p 64 and appendix p 204) are also valid for this research. From our point of view the potentially largest uncertainties in the here presented estimations may result from the extrapolation steps. Namely, the application of factors used for converting the number of BSIs to other types of infection bares uncertainties, as the daily prevalences from the PPS [9] used in this study are depending on the day of the measurement and data originate from a different geographical area (EU/EEA countries). In addition, PPS incidences were estimated by the Rhame–Sudderth formula. Not all data which were necessary for these calculations (specifically the length of stay for all patients) could be acquired from the PPS itself i.e. data from a survey of the previous year had to be used. A future improvement and potentially less bias-prone approach would be to use a method that is not dependent on any assumptions about the underlying parameter distributions [31,32]. Such an approach (i.e. a Grenander estimator) has already been used in a sensitivity analysis of the 2016–2017 PPS [9]. Additional uncertainties in DALYs’ estimates result from the application of disease models (outcome trees) which are based on published literature.

Another limitation was that no data were available on age-dependent hospitalisation days per hospital type. It was therefore not possible to calculate age-specific incidence rates. Furthermore, attributing epidemiological trends to control measures presented a challenge. Switzerland has a federal structure, and putting in place many measures proposed nationally is the responsibility of the cantonal authorities. As a result, implementation over the country is often a gradual, with timing of effects if any, difficult to pinpoint.

One important strength of this work lies in the quality and quantity of surveillance data from Swiss hospitals providing different levels of care over a period of 10 years. In particular, during the last years of the study, the number of BSI infections are highly reliable as data of all isolates from university hospitals and around 85% from non-university hospitals were collected. As a result, it was possible to robustly stratify by hospital type and linguistic region.

Finally, this study clearly demonstrates the value of analysing routinely collected data, from the individual patient to the national level. The ability to determine and follow sources and trends of antimicrobial resistance can provide crucial decision support to the development of treatment regimens as well as the design of local and national interventions. Thus, through this type of analysis the Swiss surveillance system may also inspire other countries that have more recently embarked on developing their surveillance systems.

There are numerous publications proposing improvements in AMR-bacteria burden estimations [28,33-35]. Some measures which can be considered as particularly beneficial within the context of this study include (i) a more complete sampling from other locations than blood (e.g. urinary tract or respiratory tract) and the direct inclusion of these data into the models, (ii) extending the estimations to pathogens such as *Neisseria gonorrhoeae* and others from the priority classes 2 and 3 of the WHO global priority list [36], (iii) a more distinct separation of hospital and community-acquired infections and an increased exploitation of the latter data, as well as (iv) the improvement of the models by integrating clinical data and the linkage of these data with corresponding outcomes (as proposed by Pezzani et al. [34]).

## Conclusion

This work shows that the burden of infections with antibiotic-resistant bacteria was steadily increasing over the last decade in Switzerland. This increase mainly originates from the ≥ 65-year-olds and is predominately attributed to third-generation cephalosporin-resistant *E. coli*, an important pathogen in the outpatient sector. A bundle of measures which were implemented into the Swiss healthcare system over the last years [7] may have kept typical highly resistant inpatient pathogens such as MRSA or carbapenem and colistin-resistant microorganisms at bay and thus, helped to maintain the overall burden at a moderate level.

As coverage rates and estimated outcomes in Switzerland differ considerably depending on the linguistic region and the hospital type, a stratification by these factors improved the overall burden estimation. Particularly in countries with low surveillance coverages, a potential overestimation of the burden might be reduced by using a stratified approach.

## Supplementary Data

1186000 Supplement
