# Supplementary material for: Associated deaths and disability-adjusted life-years caused by infections with antibiotic-resistant bacteria in Switzerland, 2010 to 2019
Source: Euro Surveill. 2023 May 18;28(20):2200532. doi: 10.2807/1560-7917.ES.2023.28.20.2200532 (PMC10197493; doi:10.2807/1560-7917.ES.2023.28.20.2200532)
Supplement: Supplement [file 22-00532_GASSER_Supplement.pdf]

Supplementary material for the Eurosurveillance research article "Associated deaths and disability-adjusted life-years caused by infections with antibiotic-resistant bacteria in Switzerland 2010-2019"

Disclaimer

This supplementary material is hosted by Eurosurveillance as supporting information alongside the article "Associated deaths and disability-adjusted life-years caused by infections with antibiotic-resistant bacteria in Switzerland 2010-2019", on behalf of the authors, who remain responsible for the accuracy and appropriateness of the content. The same standards for ethics, copyright, attributions and permissions as for the article apply. Supplements are not edited by Eurosurveillance and the journal is not responsible for the maintenance of any links or email addresses provided therein.

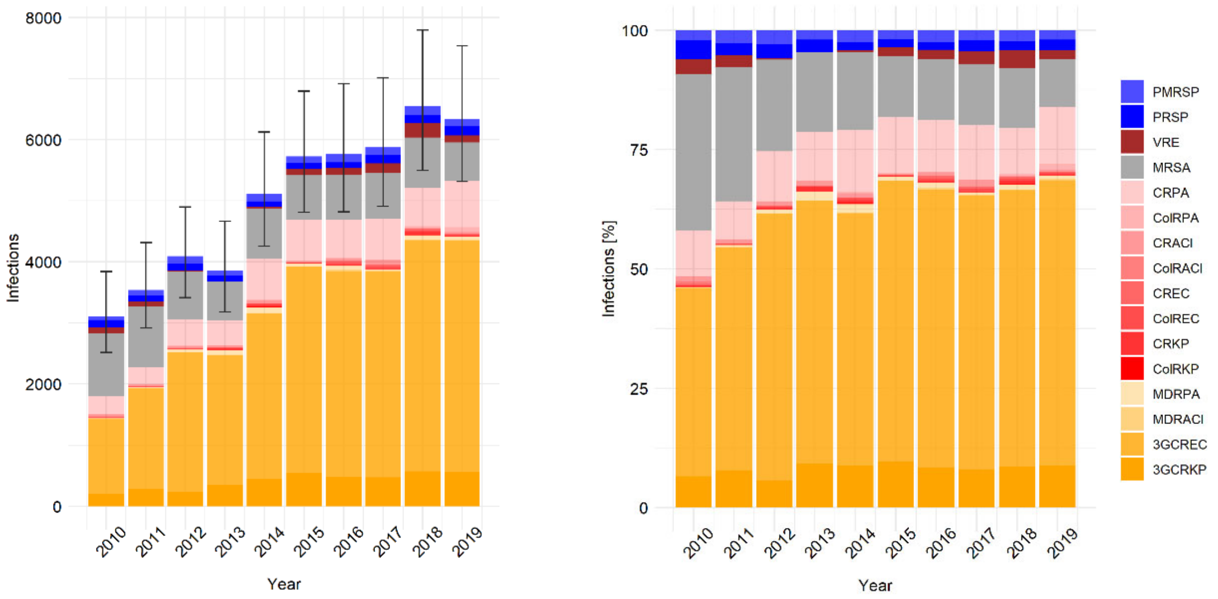

Figure S1: Infections with antibiotic-resistant bacteria in Switzerland from 2010 to 2019. Absolute values are shown on the left panel, relative contributions (%) of individual antibiotic resistant bacteria on the right panel. Data which were stratified according to the linguistic region and the hospital type were aggregated for these estimations. The 95% uncertainty intervals of the yearly totals are shown as black bars. ColRACI=colistin-resistant *Acinetobacter* spp. CRACI=carbapenem-resistant *Acinetobacter* spp. MDRACI=multidrug-resistant *Acinetobacter* spp. VRE=vancomycin-resistant *Enterococcus faecalis* and *Enterococcus faecium*. ColREC=colistin-resistant *Escherichia coli*. CREC=carbapenem-resistant *E coli*. 3GCREC=third-generation cephalosporin-resistant *E coli*. ColRKP=colistin-resistant *Klebsiella pneumoniae*. CRKP=carbapenem-resistant *K pneumoniae*. 3GCRKP=third-generation cephalosporin-resistant *K pneumoniae*. ColRPA=colistin-resistant *Pseudomonas aeruginosa*. CRPA=carbapenem-resistant *P aeruginosa*. MDRPA=multidrug-resistant *P aeruginosa*. MRSA=meticillin-resistant *Staphylococcus aureus*. PRSP=penicillin-resistant *Streptococcus pneumoniae*. PMRSP=penicillin-resistant and macrolide-resistant *S pneumoniae*.

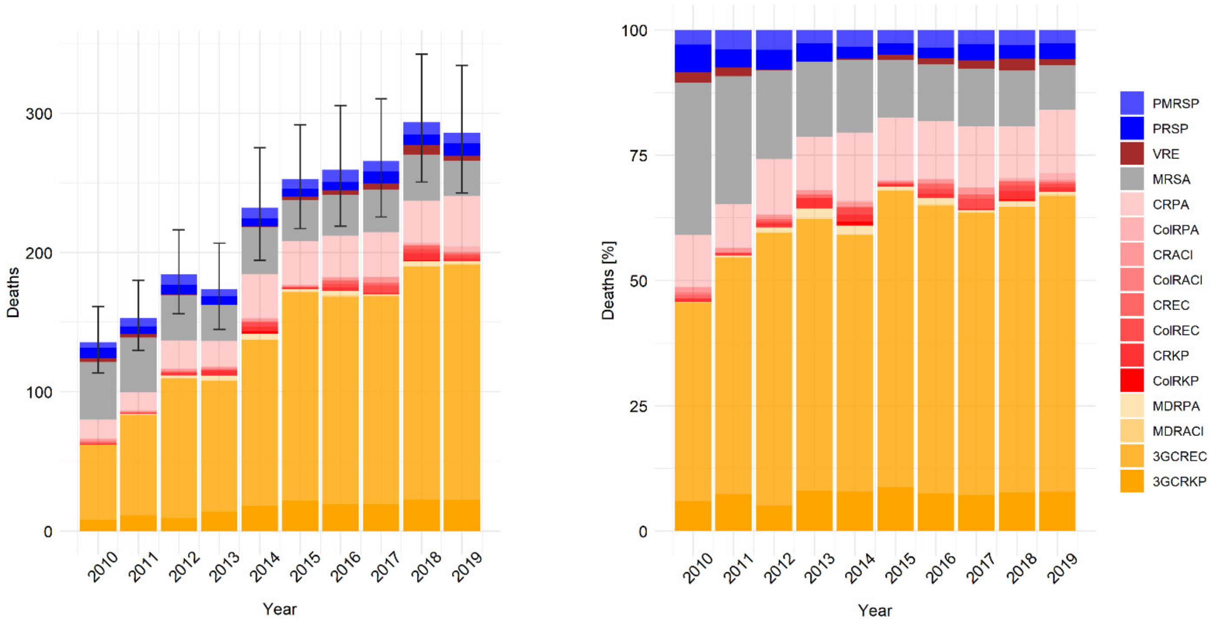

Figure S2: Deaths caused by infections with antibiotic-resistant bacteria in Switzerland from 2010 to 2019. Absolute values are shown on the left panel, relative contributions (%) of individual antibiotic resistant bacteria on the right panel. Data which were stratified according to the linguistic region and the hospital type were aggregated for these estimations. The 95% uncertainty intervals of the yearly totals are shown as black bars.

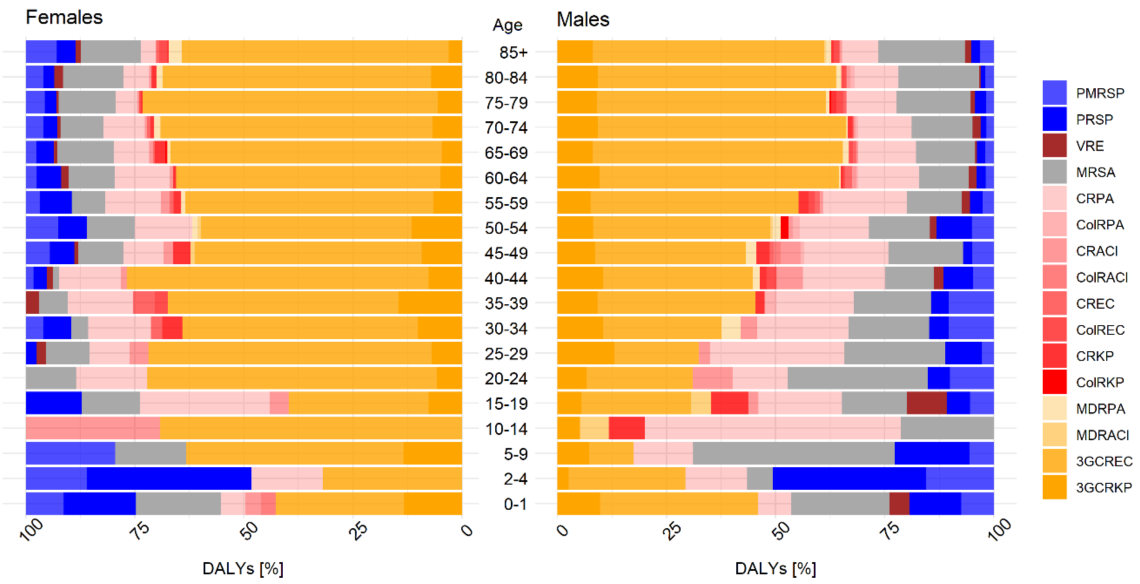

Figure S3: Proportions of DALYs in Switzerland (2010-2019) stratified the sex and age.

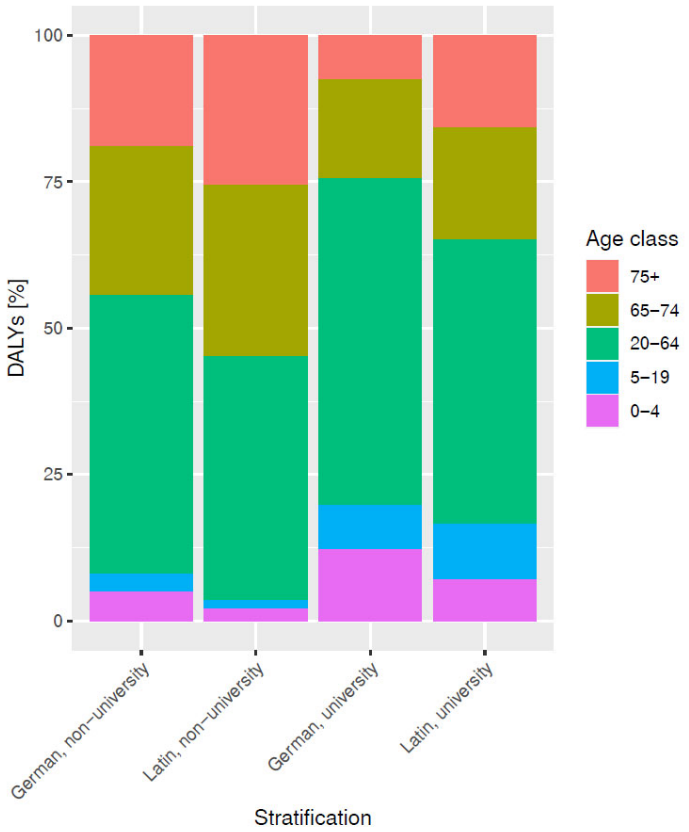

Figure S4: Age-specific DALYs distributions stratified by linguistic region and hospital type, Switzerland, 2010-2019.

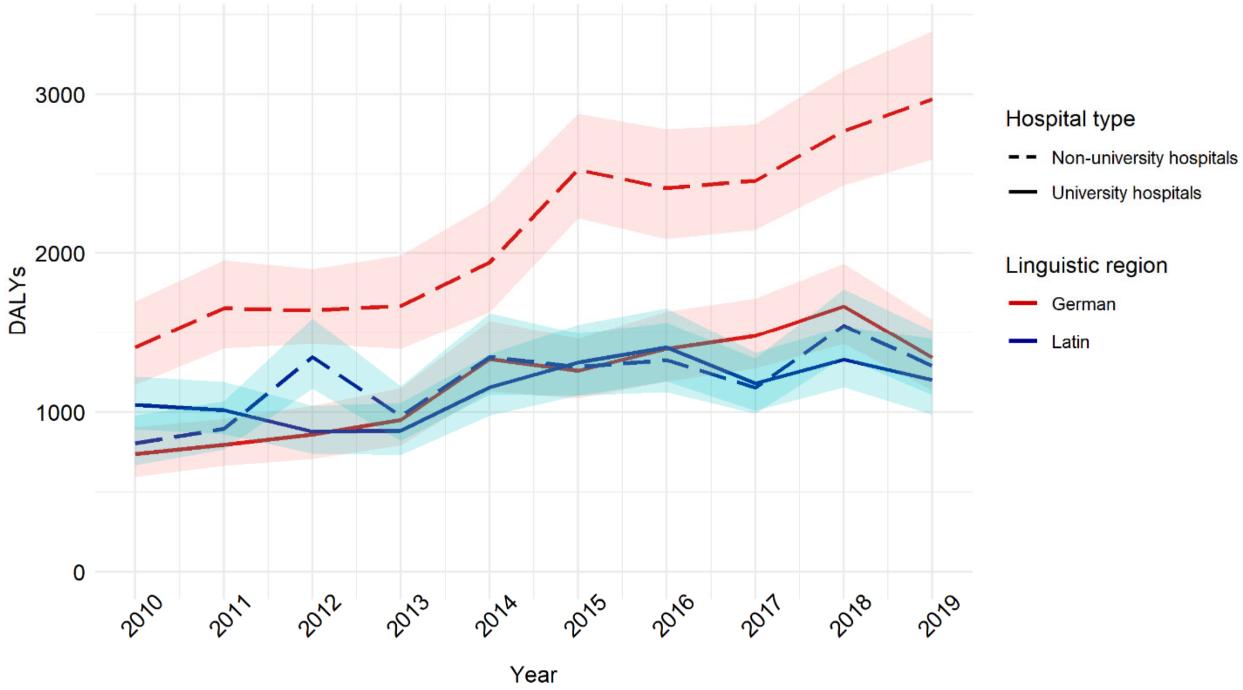

Figure S5: Model estimates of the burden of infections with antibiotic-resistant bacteria of public health importance in DALYs by linguistic region and hospital type, Switzerland, 2010-2019. The 95% uncertainty intervals are depicted by semi-transparent ribbons.

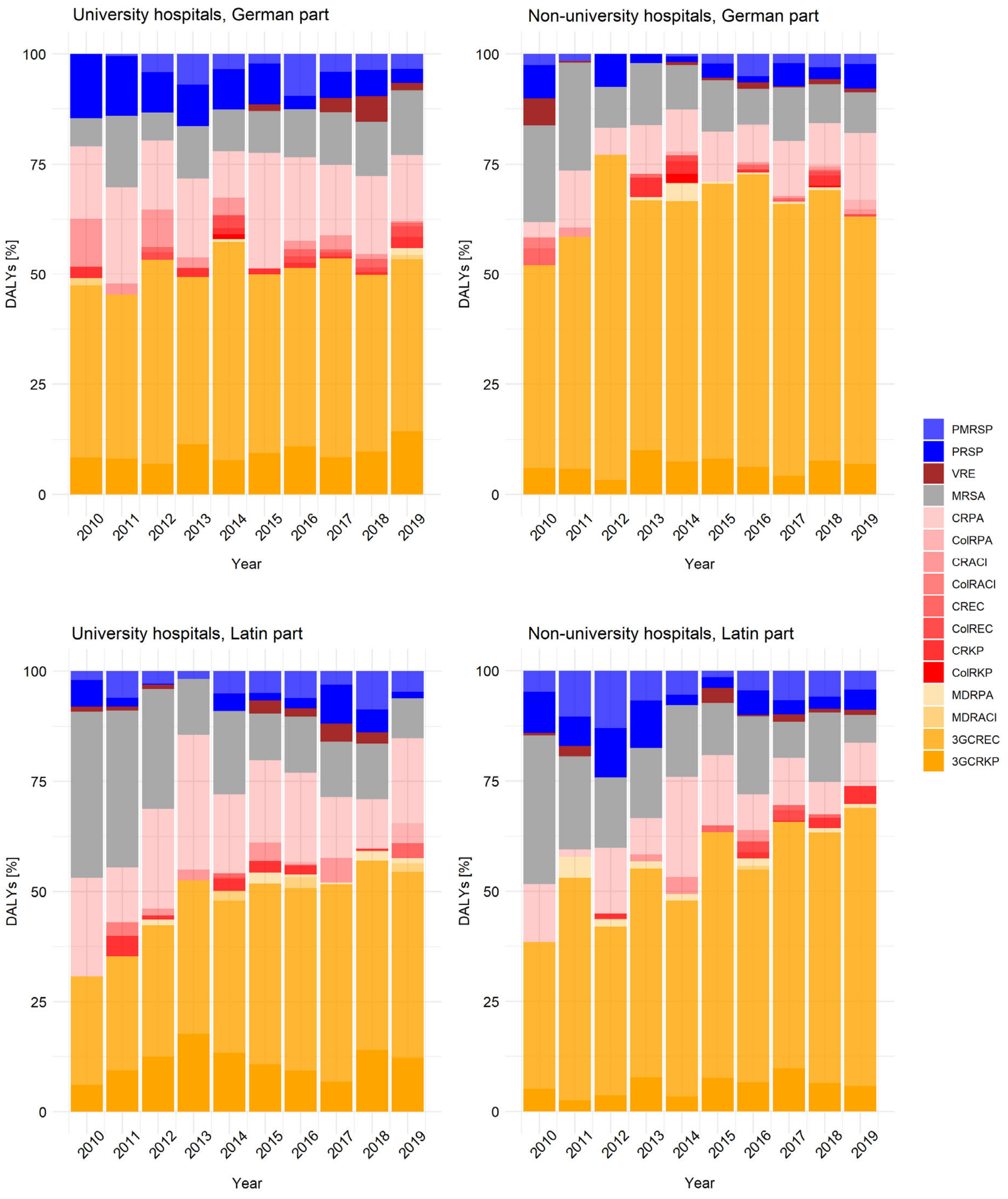

Figure S6: Proportions of DALYs in Switzerland (2010-2019) stratified by the linguistic region and the hospital type (university versus non-university hospitals).

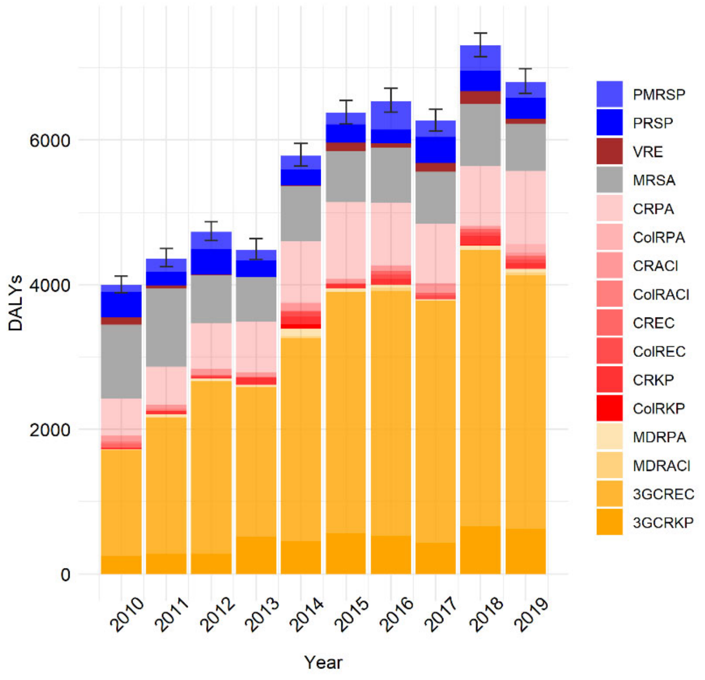

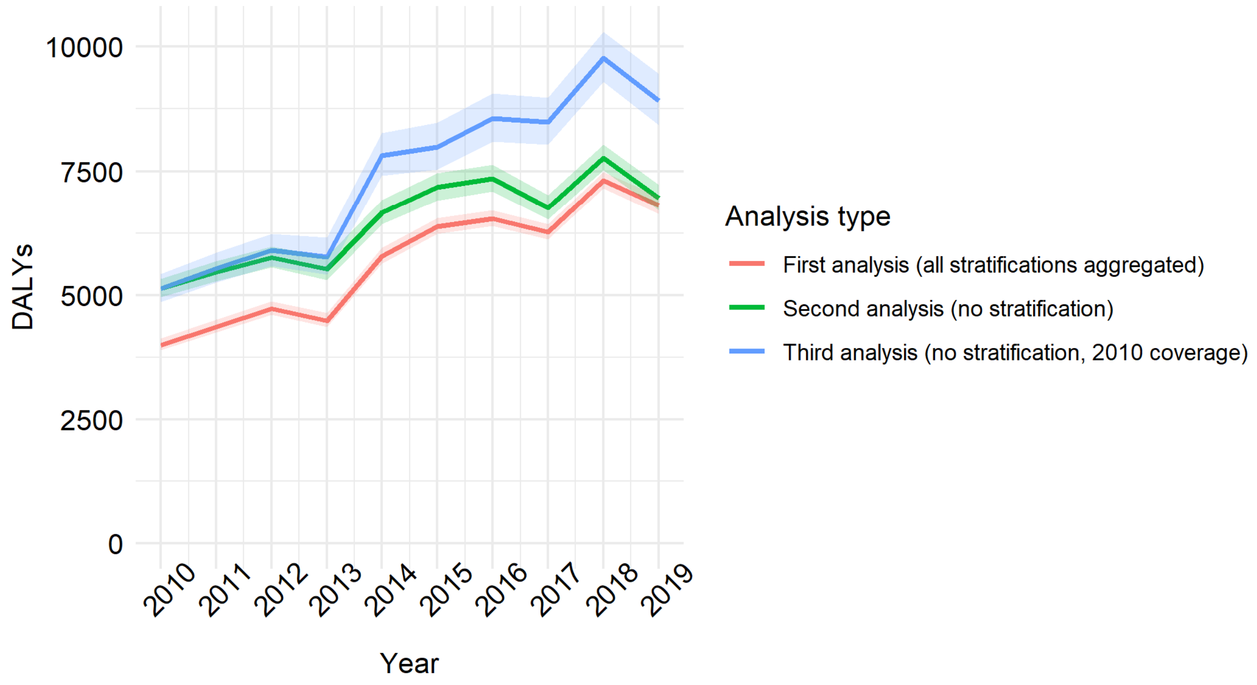

Figure S7. Representation of Figures 1A (upper panel) and 5 (lower panel) of the main manuscript using an approach assuming no correlation between sampled values when aggregating 95% uncertainty intervals of different distributions. The 95% uncertainty intervals are depicted by black bars and semi-transparent ribbons respectively.

Table S1. Yearly hospitalisation days of hospitals providing data to ANRESIS ("Hospitalisation days covered"), hospitals nationwide ("Hospitalisation days total"), and coverage rates stratified by the hospital type and the linguistic region.

Year	University						Non-university					
	German			Latin			German			Latin		
	Hospitali-sation days covered	Hospitali-sation days total	Cover-age	Hospitali-sation days covered	Hospitali-sation days total	Cover-age	Hospitali-sation days covered	Hospitali-sation days total	Cover-age	Hospitali-sation days covered	Hospitali-sation days total	Cover-age
2010	814’296	861’007	95%	545’428	545’428	100%	1’742’936	4’438’092	39%	783’821	1’379’383	57%
2011	858’392	858’392	100%	543’165	543’165	100%	1’730’636	4’417’646	39%	805’652	1’404’404	57%
2012	790’435	790’435	100%	550’548	550’548	100%	1’623’631	4’101’053	40%	770’806	1’335’994	58%
2013	803’027	803’027	100%	547’470	547’470	100%	1’653’874	4’076’994	41%	757’422	1’316’725	58%
2014	773’058	773’058	100%	569’678	569’678	100%	2’257’483	4’003’298	56%	735’488	1’313’951	56%
2015	777’908	777’908	100%	589’105	589’105	100%	2’407’634	4’088’192	59%	731’216	1’311’573	56%
2016	795’887	795’887	100%	623’446	623’446	100%	2’497’406	4’099’709	61%	920’169	1’306’764	70%
2017	855’670	855’670	100%	627’373	627’373	100%	2’812’299	4’005’810	70%	900’016	1’249’549	72%
2018	853’946	853’946	100%	623’081	623’081	100%	2’994’892	3’991’734	75%	1’063’791	1’254’693	85%
2019	861’796	861’796	100%	641’387	641’387	100%	3’369’330	3’916’628	86%	1’100’109	1’296’927	85%

Table S 2. Annual mean and median of hospitalization days per hospital (only hospitals providing data to ANRESIS).

Year	Hospitalisation days university hospitals		Hospitalisation days non-university hospitals	
	Mean	Median	Mean	Median
2010	194’246	229’717	36’620	28’835
2011	175’195	218’735	37’298	29’246
2012	167’623	192’077	35’212	27’323
2013	168’812	199’104	33’962	27’413
2014	167’842	201’326	33’255	25’756
2015	170’877	202’361	33’392	24’807
2016	177’417	205’729	31’940	22’112
2017	185’380	235’403	31’460	22’768
2018	184’628	235’755	30’064	22’344
2019	187’898	237’423	29’022	20’097

Table S3. Estimated annual burden of infections with selected antibiotic-resistant bacteria of public health importance, Switzerland, 2010-2019. ColRACI=colistin-resistant *Acinetobacter* spp. CRACI=carbapenem-resistant *Acinetobacter* spp. MDRACI=multidrug-resistant *Acinetobacter* spp. VRE=vancomycin-resistant *Enterococcus faecalis* and *Enterococcus faecium*. ColREC=colistin-resistant *Escherichia coli*. CREC=carbapenem-resistant *E coli*. 3GCREC=third-generation cephalosporin-resistant *E coli*. ColRKP=colistin-resistant *Klebsiella pneumoniae*. CRKP=carbapenem-resistant *K pneumoniae*. 3GCRKP=third-generation cephalosporin-resistant *K pneumoniae*. ColRPA=colistin-resistant *Pseudomonas aeruginosa*. CRPA=carbapenem-resistant *P aeruginosa*. MDRPA=multidrug-resistant *P aeruginosa*. MRSA=meticillin-resistant *Staphylococcus aureus*. PRSP=penicillin-resistant *Streptococcus pneumoniae*. PMRSP=penicillin-resistant and macrolide-resistant *S pneumoniae*. NA=not applicable (for small sample sizes (<60) no percentage increase calculated).

**Antibiotic-resistant bacteria**	**Infections**			**DALYs**			**Deaths**		
	**2010**	**2019**	**Increase [%]**	**2010**	**2019**	**Increase [%]**	**2010**	**2019**	**Increase [%]**
**3GCREC**	1228 (1056-1418)	3794 (3303-4326)	209	1461 (1294-1634)	3511 (3187-3851)	140	54 (47-61)	169 (149-189)	NA
**3GCRKP**	204 (163-250)	556 (484-636)	173	252 (221-287)	622 (572-675)	147	8 (7-9)	22 (21-25)	NA
**ColRACI**	16 (9-25)	0 (0-0)	NA	34 (16-56)	0 (0-0)	NA	1 (0-1)	0 (0-0)	NA
**ColREC**	0 (0-0)	17 (11-25)	NA	0 (0-0)	48 (28-68)	NA	0 (0-0)	2 (1-3)	NA
**ColRKP**	0 (0-0)	0 (0-0)	NA	0 (0-0)	0 (0-0)	NA	0 (0-0)	0 (0-0)	NA
**ColRPA**	0 (0-0)	79 (50-117)	NA	0 (0-0)	119 (69-181)	NA	0 (0-0)	4 (2-6)	NA
**CRACI**	33 (24-43)	28 (19-38)	NA	80 (52-109)	40 (23-59)	NA	2 (1-2)	1 (1-2)	NA
**CREC**	13 (8-21)	16 (10-23)	NA	53 (32-76)	51 (34-69)	NA	1 (1-1)	1 (1-2)	NA
**CRKP**	8 (5-11)	21 (14-29)	NA	20 (13-27)	87 (57-118)	NA	1 (1-1)	2 (1-3)	NA
**CRPA**	297 (206-413)	756 (580-964)	154	510 (333-739)	1012 (735-1348)	98	14 (9-20)	36 (26-48)	NA
**MDRACI**	6 (4-10)	19 (11-31)	NA	12 (6-20)	39 (18-62)	NA	0 (0-0)	1 (0-2)	NA
**MDRPA**	0 (0-0)	39 (20-71)	NA	0 (0-0)	45 (19-85)	NA	0 (0-0)	2 (1-3)	NA
**MRSA**	1020 (839-1226)	632 (519-760)	-38	1022 (898-1169)	656 (581-742)	-36	41 (36-47)	25 (22-29)	NA
**PMRSP**	64 (55-81)	125 (109-150)	94	95 (79-121)	222 (193-265)	134	4 (3-5)	8 (7-9)	NA
**PRSP**	121 (103-148)	144 (126-170)	19	352 (302-430)	288 (253-338)	-18	7 (6-9)	9 (8-10)	NA
**VRE**	100 (43-200)	116 (60-196)	16	101 (81-137)	66 (52-86)	-35	3 (2-4)	3 (3-5)	NA
**Overall**	**3110 (2516-3844)**	**6342 (5316-7538)**	**104**	**3995 (3327-4805)**	**6805 (5820-7949)**	**70**	**136 (114-161)**	**286 (243-335)**	**111**
